# Minimally invasive treatment of pediatric head and neck dermoids: percutaneous drainage and radiofrequency coblation

**DOI:** 10.1007/s00247-019-04438-w

**Published:** 2019-06-04

**Authors:** Madeline I. Foo, Leah E. Braswell, Lacey J. Lubeley, James W. Murakami

**Affiliations:** Department of Radiology, Nationwide Children’s Hospital, 700 Children’s Dr., Columbus, OH 43205 USA

**Keywords:** Children, Dermoid, Minimally invasive, Percutaneous drainage, Radiofrequency coblation

## Abstract

**Background:**

Dermoids are common benign head and neck cysts in children containing a variety of different skin elements. Current standard treatment is surgical removal that sometimes requires extensive dissection to ensure complete resection and often leaves unwanted facial scarring. A minimally invasive treatment alternative should offer a similar rate of success with a decrease in operative complexity, recovery time and postoperative scarring.

**Objective:**

To assess the outcomes of our minimally invasive percutaneous treatment of head and neck dermoids, we reviewed our 9-year interventional radiology (IR) department experience.

**Materials and methods:**

The medical records, imaging and procedural details were reviewed from a cohort of pediatric patients with dermoids treated in our IR department from January 2009 through February 2018. Patients in the study underwent ultrasound (US)-guided cyst puncture, 3% Sotradecol (sodium tetradecyl sulfate [STS]) emulsification of the thick cyst contents allowing complete drainage, and radiofrequency coblation of the cyst wall.

**Results:**

In this retrospective study, we report on 22 dermoids in 21 patients. The average patient age was 3 years. Twenty-one of the 22 dermoids were successfully treated for an overall success rate of 95%. Four intraosseous dermoids were successfully treated using computed tomography (CT) guidance instead of, or in addition to, US. Average follow-up time was 22 months.

**Conclusion:**

The combination of percutaneous cyst drainage using STS as an emulsifying agent followed by radiofrequency coblation is a safe, effective, minimally invasive treatment for pediatric patients with head and neck dermoids.

## Introduction

Dermoids are benign cysts containing skin elements such as hair follicles, sweat glands, sebaceous glands and keratinous debris [1–5]. They are most commonly diagnosed in childhood and are believed to be the result of faulty embryology [1]. Seven percent of dermoids are located in the head and neck, with the majority in the periorbital, perinasal and temporal regions [6]. They have a characteristic clinical presentation usually allowing diagnosis with history and physical exam alone though sometimes ultrasound (US) is employed to clarify questionable cases [6]. Additional imaging such as computed tomography (CT) and magnetic resonance imaging (MRI) assists lesion characterization especially to exclude intraosseous or intracranial extension in glabellar and temporal dermoids [6–8].

Standard treatment for dermoids is complete surgical excision, preferably without spillage of any cyst contents [1]. Most dermoids are near the surface of the skin allowing relatively easy removal, while dermoids that extend into the subjacent bone or orbit are more difficult to diagnose and treat [4, 5, 7]. Failure to treat these deeper dermoids can be complicated by skeletal distortion or infection [3].

While surgical resection has a high success rate, it comes with some procedural risks and potentially large facial or scalp scars [9]. A commonly overlooked consequence of facial surgery is the negative psychological impact large facial scars can have on patients [10]. Cosmetic concerns have encouraged surgeons to modify surgical techniques to make them less invasive with endoscopic techniques being the most recent advancement [9, 11].

Endoscopic surgery involves placing one or two incisions frontally or temporally, usually posterior to the hairline, followed by dissection beneath the galea to avoid branches of the facial nerve [11]. A camera is inserted through one of the incisions and working instruments to remove the dermoid through the second [11].

Other less invasive options, such as sclerotherapy, have been attempted [12, 13]. Unfortunately, these reports are very small case series. Our own experience with this technique has proved disappointing; recurrence was more the rule than the exception.

In order to increase the efficacy of our image-guided percutaneous techniques, we added radiofrequency coblation for more aggressive ablation of the cyst wall. Radiofrequencty coblation is known to be a quick, minimally invasive, and effective tissue dissection and cautery tool, which leads to permanent ablation of soft tissues contacted by the radiofrequency wand [14, 15]. Radiofrequency coblation has shown value in dermatological surgeries, head and neck open and endoscopic surgical procedures, and as a general means of controlling intraoperative bleeding [14, 16, 17].

We present our results using a combination of STS emulsification and drainage of cyst contents coupled with radiofrequency coblation of the cyst wall to treat head and neck dermoids.

## Materials and methods

Institutional Review Board (IRB) approval was obtained for this retrospective study; formal informed consent was not required.

Electronic medical records and imaging archives were reviewed from all patients who received percutaneous drainage and radiofrequency coblation of head and neck dermoids at our institution between January 2009 and February 2018. For the purpose of this study, inclusion criteria required that patients were children 18 years of age or younger, had imaging proof of diagnosis before treatment, and had follow-up for at least 3 months after the last treatment. We treated a total of 26 dermoids in pediatric and adult patients during this time period. Four dermoid treatments were excluded from this study: 2 patients (ages 27 and 29 years old) exceeded the age criterion and 2 were lost to follow-up. Our final cohort, therefore, consisted of 21 patients who had 22 dermoids. One patient had two separate dermoids treated at different times. There were four patients with intraosseous disease; two glabellar, one midline forehead and one temporal. At the time of diagnosis, none of the intraosseous dermoids was causing any symptoms. We recorded patient age, gender, locations and sizes of dermoid cysts, follow-up intervals, outcomes and any complications (Table 1).

**Table 1:** Patient demographics and outcomes

Patient	Age at first procedure (months)	Gen-der	Location**	Maximal diameter (cm)	# of treatments	Follow-up after last treatment (months)	Imaging at last follow-up	Recurrence at last follow-up	Complications
1	22	F	Left temporal	0.8	1	13	US	No	No
2	51	M	Midline suprasternal	1.3	1	31	US	No	No
3	23	F	Left periorbital	1.2	1	7	None	No	No
4	12	F	Right periorbital	1	1	19	CT	No	No
5	117	M	Midline floor of mouth	2.7	2	43	US	Yes. Dermoid removed surgically	No
6	4	M	Left periorbital	1.2	2	3	US	No	No
7	8	M	Left post auricular	0.8	1	40	US	No	No
8	9	M	Midline glabellar	0.9	1	78	US	No	No
9	11	F	Midline glabellar dermal and skull	1.2	2	34	MR	No	Post-treatment skin incision infection
10	173	M	Midline suprasternal	1.5	1	29	US	No	No
11	7	F	Right reriorbital	1	1	3	US	No	No
12	4	F	Right periorbital	1	1	18	US	No	No
13	26	M	Right temporal dermal and skull	1	2	23	CT	No	No
14*	14	F	Midline forehead	0.8	1	19	US	No	No
	27	F	Right temporal	0.7	1	7	US	No	No
15	60	F	Midline forehead skull	1	1	20	CT	No	No
16	40	M	Midline suprasternal	1	1	5	US	No	No
17	48	F	Midline suprasternal	1	1	15	US	No	No
18	33	F	Midline forehead	0.7	1	3	None	No	No
19	66	F	Midline glabellar skull	0.8	1	24	CT	No	No
20	8	F	Left periorbital	0.6	1	31	US	No	No
21	29	M	Left periorbital	1.4	1	20	None	No	No

* Patient 14 had two separate dermoids treated at different times

** Locations of dermoids were all subcutaneous except where listed as involving the skull

*CT* computed tomography, *F* female, *M* male, *US* ultrasound

**Fig. 1:**
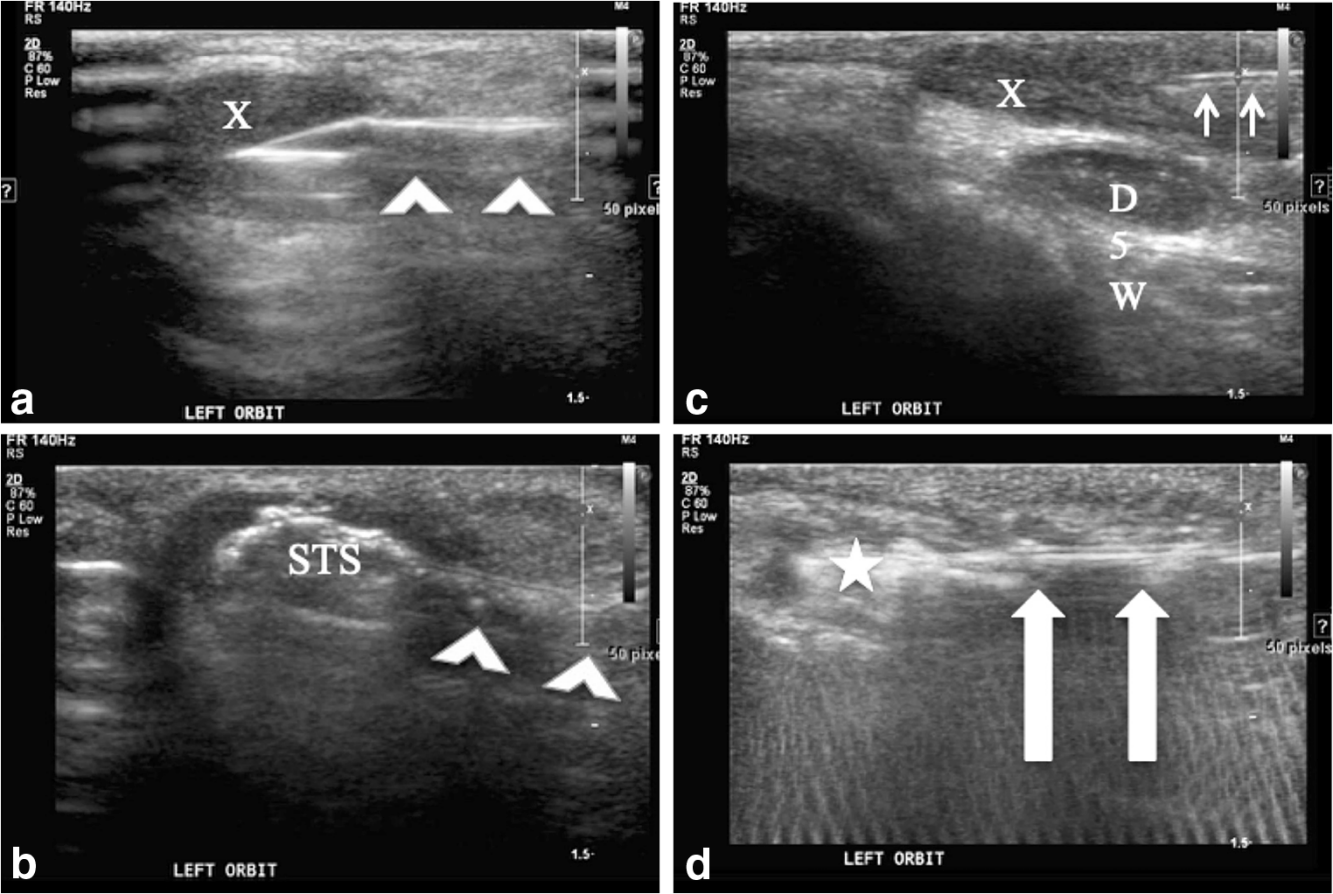
Ultrasound images during periorbital dermoid treatment in a 23-month-old girl. **a** A 14-G angiocatheter (*arrowheads*) enters the ovoid hypoechoic subcutaneous dermoid (X). **b** Echogenic sodium tetradecyl sulfate (STS) is injected centrally into the dermoid through angiocatheter (*arrowheads*) to emulsify the dermoid contents allowing aspiration. **c** A 25-G needle (*arrows*) injects 5% dextrose in water (D5W) between the dermoid and the skin surface. **d** A radiofrequency coblation wand (*arrows*) is placed through the angiocatheter to ablate the dermoid walls. Ablation can be seen as striking echogenic bubbling (*star*) and parallel echogenic linear artifacts across the image caused by the radiofrequency wand

All procedures were performed in the IR suite by two attending interventional radiologists. All procedures were performed on an outpatient basis under general anesthesia after obtaining procedural informed consent. All soft-tissue dermoids were treated using US guidance, reserving CT guidance for the four intraosseous lesions. In keeping with standard surgical practice, when possible, skin entry sites were chosen to allow access to the dermoids and to place puncture site scars in the eyebrows or behind the hairline. After making a 3-mm skin incision with a scalpel, a 14 G×1.25-in angiocatheter (Braun, Melsungen, Germany) was advanced into the cyst under image guidance (Fig. 1). Cyst contents can rarely be aspirated given their viscous, debris-laden, oily nature. Serial (3–5 times) lavage of the cyst with small aliquots of 3% sodium tetradecyl sulfate (STS) (Mylan Institutional LLC, Rockford, IL) will result in complete drainage of most cysts (Fig. 1). The lavage is accomplished with a 1- or 3-ml syringe containing 0.2–1.0 ml of STS that is used to inject the STS into the angiocatheter under direct US guidance. Several minutes later, when the STS is aspirated, the thick white keratinous debris will be removed with the STS. This process needs to be repeated until, by US, the cyst is emptied of its contents.

A 25-G needle was placed under US guidance superficial to the cysts to inject 2–5 ml of 5% dextrose in water (D5W) around the cyst to insulate the skin surface from any radiofrequency energy (Fig. 1). Unlike cryoablation, where saline can be used to distance adjacent tissue from freezing damage, when using radiofrequency techniques a nonionic solution such as D5W needs to be used to insulate adjacent tissue from ion agitation and heating. A 17-G radiofrequency coblation wand (Perc DLR Spinewand; ArthroCare Corp., Austin, TX) was then advanced through the 14-G angiocatheter under image guidance and used to coblate the walls of the cyst over 2–3 min using the higher settings on the radiofrequency generator of three or four out of four levels (Fig. 1). After removing the wand, any remaining cyst contents were manually expressed out the entry incision. No entry incisions were sutured. Some were closed with tape, but most were simply covered with a standard dressing. All patients were discharged to home from our post-anesthesia care unit once discharge criteria were met. Postoperative prophylactic antibiotics were given for 7 days. The 7-day course of antibiotics was chosen to cover the time frame of potential inflammation caused by the procedure and may be more than what is needed for infection prophylaxis.

Outcomes were assessed by physical exam and imaging in all patients but three for whom only phone follow-up could be obtained. Follow-up times ranged from 3 months to 78 months, with a mean of 22 months. While an attempt was made to have follow-up clinic appointments between 3 and 6 months, many times this was not achieved and the follow-up times reflect when the patients could be contacted. US was used at all clinical follow-up appointments. Usually a small fibrous scar or nothing can be seen with US in the area of a treated dermoid. CT and MRI were also used for patients with intraosseous extension. If the dermoid recurred during the follow-up time period, the treatment was repeated a second time using identical procedural technique. When recurrent, a dermoid is easily palpable and visible as a cyst by US. When a cyst recurred, a second treatment was scheduled at the next convenient time for the patient. Success was defined as complete clinical and imaging resolution during the follow-up time period. Failure was defined as recurrence of any part of the cyst after two treatments. Any complications were recorded.

## Results

**Fig. 2:**
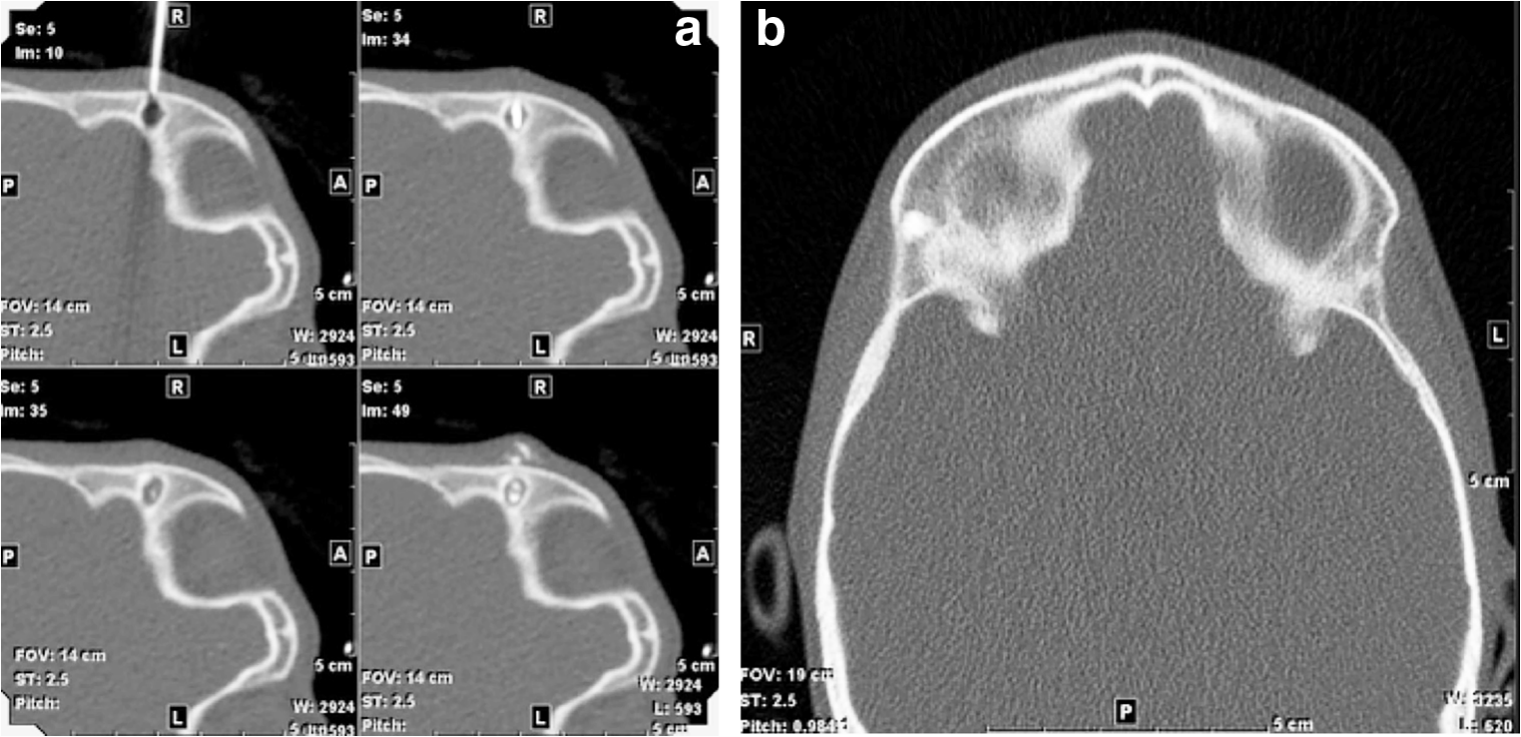
A right lateral frontal dermoid with extension into the subjacent bone treated with CT guidance in a 32-month-old boy. **a** Four frames from the CT-guided radiofrequency coblation show the radiofrequency coblation wand embedded in the lesion during ablation. Intravenous contrast had been injected through the guiding catheter to exclude intracranial extension and can be seen in the intraosseous portion of the lesion and the overlying soft tissues. **b** Follow-up CT obtained 23 months later shows that the entirety of the lesion is densely ossified and healed

Treatment outcomes for all cases are detailed in Table 1. The age range of our cohort was 4 months to 14 years with a mean of 36 months. Twenty-one of the 22 dermoids were successfully treated. The four patients with intraosseous disease were treated using CT guidance instead of US and their dermoids were all successfully ablated (Fig. 2). Eighteen dermoids were successfully treated with one procedure. Three dermoids required two treatments and two of these were intraosseous lesions. Recurrent or residual dermoids were all identified at the first clinical follow-up visit or imaging session (between 3 and 7 months). Subcutaneous recurrences were clinically apparent and visible with US. The two cases with residual/recurrent osseous lesions were identified on the first post-treatment CT scans. Three subcutaneous dermoids had no imaging follow-up with outcomes assessed by phone follow-up. One large, 2.7-cm diameter, floor-of-mouth dermoid was treated and recurred twice and was subsequently surgically removed.

There was one procedural complication, a postoperative skin infection occurring several days after treatment of an intraosseous glabellar dermoid. This infection was successfully managed by small subcutaneous abscess aspiration and a single course of oral antibiotics. In 16 cases, cyst contents were sent to pathology where evaluation confirmed the diagnosis of a dermoid with the primary finding of keratin debris and occasional hair shafts and squamous cells.

## Discussion

Head and neck dermoids are a common pediatric problem familiar to most pediatricians, surgeons and radiologists. The exact cause of these dermoids is not known, but the most common theory is that deeper tissues fail to properly fuse during early embryonic life leading to epithelial rests that later form debris-filled cysts [1, 5, 8].

Imaging is often not needed when making the diagnosis of a dermoid. However, because we have US readily available in our clinic and it is quick and easy to perform, we use it routinely for patient evaluation, family education and treatment planning. Deeper and more complex dermoids often need CT and MRI to assist in lesion characterization to properly diagnose intraosseous or intracranial extension in glabellar or temporal dermoids [2, 3, 6, 7, 18].

Diagnostic confirmation can be obtained by pathological evaluation of extracted material. In our study, we sent material to pathology in 16 out of 22 cases. Each dermoid had thick white cheese-like cyst contents, which pathology reports confirmed to be keratin and consistent with the diagnosis. After consistently receiving identical biopsy reports, we found that the clinical presentation and imaging, together with the gross appearance of aspirated material, is sufficient for complete diagnosis and sending cyst contents for laboratory evaluation is unnecessary.

Failure to treat dermoids can result in skeletal distortion or infections [3]. Complete surgical removal is the current standard treatment and has low recurrence rates [1, 2, 4, 6]. However, failure to remove the entire cyst carries a 50–100% recurrence rate [3, 19]. Although the majority of head and neck pediatric dermoids are small and superficial, surgical resection has been reported to have an 18% risk of intraoperative cyst rupture [8]. These ruptures can lead to inflammation, fistula formation and an increased risk of cyst recurrence [8, 11, 19].

Surgical removal of dermoids with intraosseous extension often requires a multidisciplinary operative team including plastic surgeons, craniofacial surgeons, neurosurgeons, ophthalmologists and otolaryngologists often mandating extensive scalp and skull dissection [2, 3, 7]. While this is often successful, the complex nature of this procedure is associated with not only expected side effects of anosmia and facial scarring but also risks such as wound infection, nerve injury and even seizures [2, 5, 9, 18]. Two studies report significant patient dissatisfaction with facial scarring and deformity from these procedures [5, 10]. Cosmetic concerns have prompted surgeons to revise their techniques to make them less invasive with the recent introduction of endoscopy [9, 11].

Endoscopic surgery has the ability to move the 10- to 15-mm-long surgical scars from cosmetically sensitive areas on the face to behind the hairline [9, 11]. Working endoscopically with more limited exposure is more prone to cyst rupture during resection, leading to more inflammation and potentially retained epidermal tissue [20].

Sclerotherapy can treat different types of cysts in the head and neck [21, 22]. STS sclerotherapy has been reported in small case series format to treat orbital and periorbital dermoids [12, 13]. Unfortunately, our clinical experience with this technique revealed recurrence to be the rule and not the exception. While we cannot discount the possibility that the STS we used for emulsification and drainage of cyst contents before radiofrequency coblation may be additive or even necessary for the success of the procedures reported here, our past experience has taught us that STS is not a sufficient stand-alone treatment in our hands.

In order to increase the success of our treatment of dermoids, we added radiofrequency coblation, an accepted surgical tool used in dermatology and otolaryngology [14]. Radiofrequency coblation is known to be an effective tissue dissection technique, commonly used during tonsillectomies and nasal turbinate reductions [16, 23].

Radiofrequency coblation ablates tissue the wand contacts with less adjacent tissue heating and damage than radiofrequency cautery [15]. The wand tip generates a radiofrequency current that passes through the ions in the target tissue, agitating them to break apart molecular bonds at relatively low temperatures (40–70°C) [14, 15]. This allows for precise surgical interventions, with limited energy penetration deeper than the surface directly contacted by the wand [15]. Injection of an ionic solution such as saline (or STS) into the target tissue or cyst before coblation will increase the energy deposition [23]. In contrast to D5W, which is injected around the cyst to protect adjacent tissues from radiofrequency energy, ionic solutions such as saline or STS injected into the cyst will enhance the radiofrequency energy deposition into the wall of the cyst.

At a temperature of 70°C, within the range caused by the radiofrequency coblation wand, epithelial cells are rapidly destroyed [24]. Deep partial thickness skin burns occur after less than a 30-s exposure to water heated in the range of 60–70°C [25]. The radiofrequency coblation wand therefore generates energies sufficient to ablate an epithelial surface such as a cyst wall within this time frame.

To limit tissue heating near the ablated area, D5W can be injected around the target tissue [26]. D5W reduces thermal damage to surrounding heat-sensitive skin and tissues by providing an insulating blanket around the targeted lesion [26]. We had no skin burns near any of our treated dermoids.

In our retrospective study we had an overall success rate of 95% -- a value comparable with those reported in surgical series [1, 4]. Three dermoids needed two treatments to achieve complete cyst resolution. Repeat treatment, when needed, was easily performed and well tolerated. Unlike operative techniques where scarring commonly makes the second procedure more difficult, repeating a percutaneous puncture is just as simple as the first, often using the same skin entry point. Two of the three dermoids requiring second treatments had intraosseous disease. All intraosseous disease patients underwent CT at a follow-up appointment to confirm complete resolution of the cyst. We followed intraosseous lesions for a longer time (mean: 26 months) to ensure solid bone healing. Treating intraosseous disease is technically more difficult because it requires CT guidance and accurate wand placement likely leading to the higher incidence of incomplete treatment in this subset of our patients. Despite the fact that two of our four cases of intraosseous disease required two treatments for ultimate success, this is the subpopulation of dermoids that requires the most complex open surgical procedures for complete resection and may, in fact, be the group of patients who benefit most from a less invasive option such as our radiofrequency coblation technique.

Our one treatment failure was a large, 2.7-cm diameter, floor-of-mouth dermoid that recurred after two treatments and was subsequently surgically removed. We believe that larger cysts are more difficult to treat with radiofrequency coblation as it is more technically challenging to successfully ablate the entire wall of a larger cyst and we no longer attempt to use this technique on cysts greater than 2 cm in diameter.

All cases with recurrent or residual disease were identified at the 3-month follow-up visit if the lesions were in the subcutaneous tissues and were identified at the first imaging follow-up for intraosseous disease by 6 months after the initial treatment. This suggests that clinical follow-up longer than 3–6 months is unlikely to find recurrent disease for subcutaneous dermoids. In addition, if a dermoid were to recur beyond this time frame, it would be easily palpable by the patient and their family. Although our number of intraosseous dermoids was only four, the CT images we got at follow-up in these patients, together with other work we have done treating benign lytic osseous disease such as aneurysmal bone cysts, would suggest a longer follow-up of several years is necessary to ensure complete osseous fill-in of treated dermoids.

All procedures were technically successful and were performed on an outpatient basis. We had one postoperative infection requiring small subcutaneous abscess aspiration and a single course of oral antibiotics. This case, together with our observation of post-treatment inflammation that can be confused with infection by family members or other providers, has altered our treatment protocol to include 7 days of antibiotics. Pain medication is not routinely prescribed after this procedure. Though we did not measure the length or conspicuity of patients’ scars, they were all small reflecting the 3-mm entry incision. Many were difficult for the families to locate during follow-up visits, and we received no complaints from any patients about scarring.

A limitation of this study is the lack of imaging follow-up for three patients with subcutaneous dermoids. Mitigating this limitation is that any recurrence of a subcutaneous dermoid would be easily discovered by patients and their families with simple palpation. Surgeons rarely get any imaging follow-up after resection of a dermoid without osseous involvement [4, 6]. As noted above, another limitation of our technique is that it is not clear what role STS plays in the ultimate success of our procedure that uses combined STS and radiofrequency coblation. Further work could try to tease out which steps are necessary for success. From a practical standpoint, we know of no other way to emulsify and drain a dermoid through a small cannula thus, for now, necessitating the use of STS.

## Conclusion

We believe that our results are sufficient to introduce percutaneous dermoid cyst drainage and radiofrequency coblation of the cyst wall as a safe, effective treatment option for head and neck dermoids in children.
